# Microarray Data of Lacrimal Gland Implicates Dysregulated Protein Processing in Endoplasmic Reticulum in Graves’ Ophthalmopathy

**DOI:** 10.3389/fendo.2020.571151

**Published:** 2021-02-03

**Authors:** Wenling Tu, Jia Yao, Zhanjun Mei, Xue Jiang, Yuhong Shi

**Affiliations:** ^1^ Department of Nuclear Medicine, The Second Affiliated Hospital of Chengdu Medical College, China National Nuclear Corporation 416 Hospital, Chengdu, China; ^2^ School of Bioscience and Technology, Chengdu Medical College, Chengdu, China; ^3^ Research and Development Center, Chengdu SuAn Technology Co., Ltd, Chengdu, China

**Keywords:** Graves’ ophthalmopathy, lacrimal gland, protein processing in endoplasmic reticulum, hub gene, microarray data

## Abstract

Graves’ ophthalmopathy (GO) has become one of the most common orbital diseases. Although some evidences announced the potential mechanism of pathological changes in extraocular muscle and orbital adipose tissue, little is known about that in lacrimal enlargement of GO patients. Thus, gene expression profiles of lacrimal gland derived from GO patients and normal controls were investigated using the microarray datasets of GSE105149 and GSE58331. The raw data and annotation files of GSE105149 and GSE58331 were downloaded from Gene Expression Omnibus (GEO) database. Bioinformatics including differentially expressed genes (DEGs), Gene Ontology, Kyoto Encyclopedia of Gene and Genome (KEGG) pathway, protein-protein interaction (PPI) network construction, hub gene identification, and gene set variation analysis (GSVA) were successively performed. A total of 173 overlapping DEGs in GSE105149 and GSE58331 were screened out, including 20 up-regulated and 153 down-regulated genes. Gene Ontology, KEGG and GSVA analyses of these DEGs showed that the most significant mechanism was closely associated with endoplasmic reticulum (ER). Moreover, we identified 40 module genes and 13 hub genes which were also enriched in the ER-associated terms and pathways. Among the hub genes, five genes including *HSP90AA1*, *HSP90B1*, *DNAJC10*, *HSPA5*, and *CANX* may be involved in the dysfunction of protein processing in ER. Taken together, our observations revealed a dysregulated gene network which is essential for protein processing in ER in GO patients. These findings provided a potential mechanism in the progression of lacrimal enlargement in GO patients, as a new insight into GO pathogenesis.

## Introduction

Graves’ ophthalmopathy (GO), also known as thyroid eye disease (TED), is an orbital disease that is uniquely linked to Graves’ disease (GD), generally present in 25%–50% of GD patients (1). The main clinical features of GO include proptosis, lacrimal gland enlargement, eyelid retraction, diplopia, and exposure keratopathy, even developing into irreversible vision loss in severe cases (2). It is generally agreed that GO is an autoimmune disease that results in orbital remodeling, enlargement and fibrosis (3). These pathological changes involves three distinct but related immune processes: inflammation, adipogenesis and glycosaminoglycan accumulation (4). Most investigators have hypothesized that orbital fibroblasts are the main targets of inflammatory cytokines released by infiltrated immune cells in the extraocular muscle and orbital adipose tissue (5). Moreover, activated orbital fibroblasts could secrete many cytokines, such as interleukins 1β, 6, 8, 16, TNF-α, RANTES and CD154 (6). These cytokines promote orbital trafficking of monocytes and macrophages, facilitate differentiation of orbital fibroblasts and stimulate accumulation of hyaluronic acid-rich stroma, leading to uncontrolled immune responses (4–6). Although several mechanisms have been proposed for the development of GO, the exact pathogenesis of GO has not yet been illustrated.

Recently, high-throughput technologies provided ample evidence on the potential biomarkers and molecular mechanisms of GO. For examples, microarray analysis revealed the potential role of CASQ2 to trigger autoimmunity events (7), and identified several adipogenesis-related genes and some genes involved in Wnt and IGF-1 signaling as being potentially implicated in pathogenesis (8–11). RNA-Seq also identified some meaningful genes (such as *PTX3*, *HOXB2*, *HOXB3*, *CCL2*, and *SERPINA1*) as potential biomarkers of GO (12–14). In addition, microRNA and protein sequencing found some blood circulating biomarkers that have the potential to diagnose GD, predict GO disease status and optimize patient management, such as hsa-miR-27a-3p, hsa-miR-22-3p, zonulin, haptoglobin, and lumican (15). All these accumulating findings improve our knowledge of the mechanisms underlying pathological changes in extraocular muscle and orbital adipose tissue of GO. Nevertheless, our understanding of the molecular mechanisms of lacrimal gland enlargement in GO is still limited.

In the present study, microarray analysis was performed to systematically investigate the key genes and pathways in lacrimal gland by mining the microarray datasets (GSE105149 and GSE58331) of GO and normal lacrimal samples. GSE105149 and GSE58331 were obtained from the public database GEO. These results would help to uncover a potential mechanism of lacrimal enlargement and offer new information for the pathogenesis of GO.

## Materials and Methods

### Characteristics of the Microarray Data Sets

The Gene Expression Omnibus (GEO, http://www.ncbi.nlm.nih.gov/geo/) is a public functional genomics data repository. GEO has collected a large amount of omics data, such as high-throughput gene expression data, chips and microarrays. Microarray datasets of GSE105149 and GSE58331 were obtained by searching the keywords including Graves’ ophthalmopathy, thyroid eye disease and thyroid-associated ophthalmopathy in GEO. The raw data and annotation files of GSE105149 and GSE58331 were downloaded from the GEO database. Rosenbaum JT et al. contributed to the generation of GSE105149 and GSE58331and provided many significant findings from different perspectives (16–21). GSE105149 was the microarray data of various lacrimal gland tissues, including sarcoidosis, granulomatosis with polyangiitis, thyroid eye disease, nonspecific orbital inflammation and healthy controls (16). Normal lacrimal samples from healthy individuals were obtained at the time of cosmetic surgery or blepharoplasty (16). GSE58331 was the microarray data of anterior orbit and lacrimal gland tissues, which were obtained from subjects with inflammatory diseases such as nonspecific orbital inflammation, sarcoidosis, granulomatosis with polyangiitis, IgG4-associated disease and thyroid eye disease as wells as from normal controls (17–21). Normal control tissues were obtained during surgeries such as blepharoplasty and enucleation on eyes with non-inflamed orbits (17–21). The diagnoses of all samples were based on the clinical and histopathological information submitted by orbital disease specialists and ocular pathologists (16–21). On account of GO or normal lacrimal gland as the inclusion criteria, the following samples were chosen in the present study: four TED lacrimal samples (GSM2823348, GSM2823349, GSM2823350, GSM2823351) and seven normal lacrimal samples (GSM2823306, GSM2823307, GSM2823308, GSM2823309, GSM2823310, GSM2823311, GSM2823312) were derived from GSE105149, and no sample was repeated; eight TED lacrimal samples (GSM1407195, GSM1407196, GSM1407199, GSM1407200, GSM1407202, GSM1407203, GSM1407204, GSM1407206) and seven normal lacrimal samples (GSM1407233, GSM1407235, GSM1407236, GSM1407237, GSM1407238, GSM1407240, GSM1407243) were obtained from GSE58331, in which eight TED samples were from four patients. Both microarrays have the same platform of GPL570 [HG-U133_Plus_2] Affymetrix Human Genome U133 Plus 2.0 Array.

### Screening of the Differentially Expressed Genes

According to the raw data and annotation files, microarray probes were converted into corresponding gene symbols, and the expression value of one gene symbol corresponding to multiple probes was calculated by taking the average value. In addition, Z-score was chosen to normalize the converted data to extend the range of differential expression. The Limma R package (http://www.bioconductor.org/packages/release/bioc/html/limma.html) was used to detect differentially expressed genes (DEGs) between TED and normal lacrimal group. The cut-off criterion of DEGs was P< 0.05 and |log (fold change) | ≥1. The false discovery rate (FDR) was not used to identify DEGs in the present study. Nonetheless, we calculated FDR and published it alongside the p-values to give a better idea of the robustness of DEGs.

### Gene Ontology and Kyoto Encyclopedia of Genes and Genomes Pathway Analyses

Using the Database for Annotation, Visualization and Integrated Discovery (DAVID, https://david.ncifcrf.gov/), DEGs were mapped to Gene Ontology analysis to detect their possible functional processes from three terms of biological process (BP), molecular function (MF) and cellular component (CC). Moreover, Kyoto Encyclopedia of Genes and Genomes (KEGG) pathway analysis of DEGs was carried out to investigate their potential biological pathways by the KEGG Orthology-based Annotation System 2.0 (KOBAS 2.0, http://kobas.cbi.pku.edu.cn). P < 0.05 was considered as statistically significant difference.

### Construction of Protein-Protein Interaction Network

DEGs were entered into the Search Tool for the Retrieval of Interacting Genes (STRING) database (https://string-db.org/) to construct a functional protein-protein interaction (PPI) network. An interaction with the composite score > 0.4 was considered statistically significant. Then, PPI network complex was visualized with Cytoscape software. The central node genes were screened out with the cut-off criteria of connectivity degree ≥10. Whereafter, the Molecular Complex Detection (MCODE) in Cytoscape software was utilized to identify densely connected regions from PPI network complex. The connected region with MCODE score >3 and node number >4 was considered to be the significant module.

### Gene Set Variation Analysis

Gene set variation analysis (GSVA) is a nonparametric and unsupervised clustering approach to estimate the score of gene-set enriched pathways based on the expression profile of each sample (22). For GSVA analysis, we chose HALLMARK gene sets as the reference, t value > 2 and p value < 0.05 as the cut-off to identify significantly altered pathways. The GSVA R package (http://www.bioconductor.org/packages/release/bioc/html/GSVA.html) was used to calculate a pathway enrichment score between TED and normal lacrimal group. GSVA analysis for GSE105149, GSE58331, the combined data of GSE105149 and GSE58331 were performed, respectively.

## Results

### Screening of the Common Molecular Origins Associated with GO Lacrimal Enlargement

**Figure 1:**
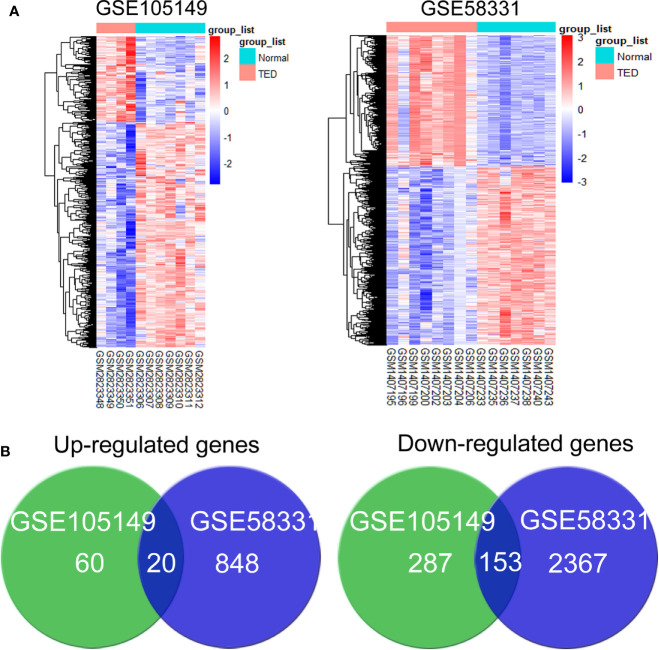
Screening of the common differentially expressed genes associated with lacrimal enlargement in thyroid eye disease (TED). **(A)** hierarchical clustering heat maps showed different expression of all differentially expressed genes (DEGs) in the TED lacrimal samples relative to normal lacrimal samples. Red indicates that the expression of genes is relatively upregulated, and blue indicates that the expression of genes is relatively downregulated. **(B)** The DEGs were identified with the P-value <0.05 and |log (fold change)≥1 between thyroid eye disease (TED) and normal lacrimal samples from the mRNA expression profiling sets GSE105149 and GSE58331. Venn diagram showed the respective frequency of up-regulated and down-regulated genes in the TED lacrimal samples.

Using P< 0.05 and |log (fold change) | ≥1 as the cut-off criterion, data from GSE105149 and GSE58331 were separately analyzed by the limma R package to identify DEGs. Representative heat maps showed different expression of all DEGs in the TED lacrimal samples relative to normal lacrimal samples (**Figure 1A**). 80 up-regulated and 440 down-regulated genes were present in the TED lacrimal samples of GSE105149, 868 up-regulated and 252 down-regulated genes were in the TED lacrimal samples of GSE58331. The overlap between the two microarray datasets contained 173 genes, including 20 up-regulated and 153 down-regulated genes in the TED lacrimal glands (**Figure 1B**, **Table S1**). Collectively, the common DEGs in two different gene expression profiles provided evidence on the common molecular origins of lacrimal gland enlargement in GO patients.

### Investigation of the Potential Functions and Pathways in GO Lacrimal Enlargement

**Figure 2:**
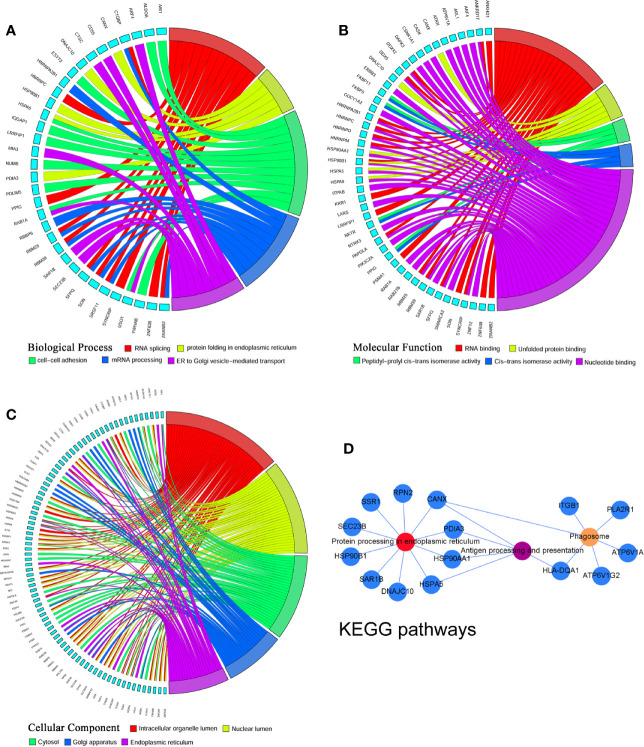
Gene Ontology terms and Kyoto Encyclopedia of Genes and Genomes (KEGG) pathways of the common differentially expressed genes (DEGs) set. Circos plot represents the relationship between genes and the top 5 significant terms including biological process **(A)** cellular component **(B)** and molecular function **(C)**. Different colors from red, yellow, green, blue to purple indicate that the P values are sorted from minimum to maximum. **(D)** KEGG pathway analysis showed that DEGs were mainly enriched in pathways related to protein processing in endoplasmic reticulum, phagosome, and antigen processing and presentation. Different colors from red, yellow to purple indicate that the P values are sorted from minimum to maximum.

To investigate the mechanism, the overlapping DEGs were further measured through Gene Ontology and KEGG pathway analyses to explore the possible functions and pathways in the pathogenesis of GO lacrimal enlargement. Based on DAVID software, we observed a total of 81 Gene Ontology assignments, including 31 BPs, 9 MFs, and 41 CCs. The top five significant terms of Gene Ontology classification were illustrated in **Figures 2A–C**. In the BP category, most DEGs were involved in RNA splicing, protein folding in ER, cell-cell adhesion, mRNA processing and ER to Golgi vesicle-mediated transport. In the MF category, most DEGs may play roles in RNA binding, unfolded protein binding, peptidyl-prolyl cis-trans isomerase activity, cis-trans isomerase activity and nucleotide binding. In the CC category, a large percentage of the DEGs were associated with intracellular organelle lumen, nuclear lumen, cytosol, Golgi apparatus and ER. Furthermore, the KEGG analysis showed the dysfunction of protein processing in ER, phagosome and antigen processing and presentation pathways in GO lacrimal samples (**Figure 2D**). Taken together, these findings suggested that multiple functions and pathways were significantly associated with GO lacrimal enlargement.

### Identification of the Most Significant Pathway and Hub Genes in GO Lacrimal Enlargement

**Figure 3:**
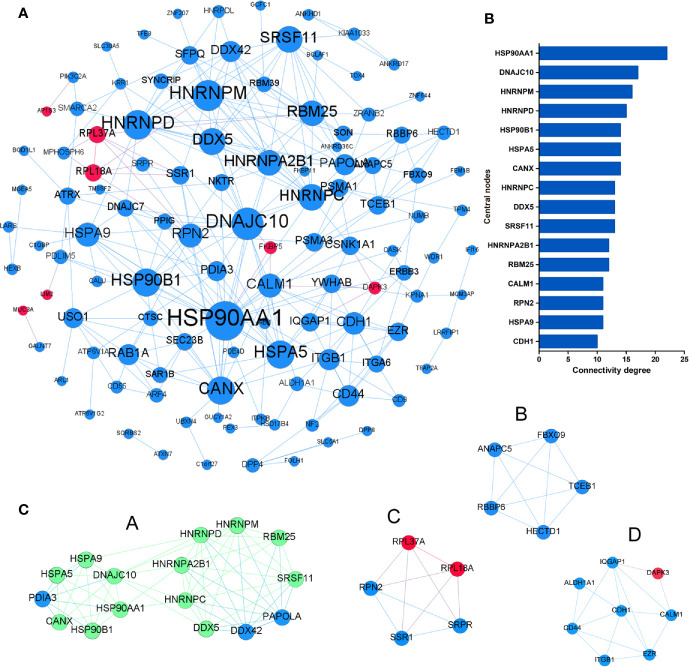
Protein-protein interaction network of the common differentially expressed genes (DEGs) set. **(A)** The protein-protein interaction (PPI) network was visualized using Cytoscape software. The node size was proportional to the connectivity degree. The genes with no connectivity were not present in the network. **(B)** A total of 16 central node genes were screened out with the criteria of filtering degree ≥ 10 from the PPI network complex. **(C)** Module a, module b, module c, and module d were regarded as significant modules with the selection criteria of MCODE score > 3 and node number > 4. Red indicates that the expression of genes is relatively upregulated, and blue indicates that the expression of genes is relatively downregulated. In the module a, green indicates that the common genes present in 16 central nodes and module a were selected as hub genes.

**Figure 4:**
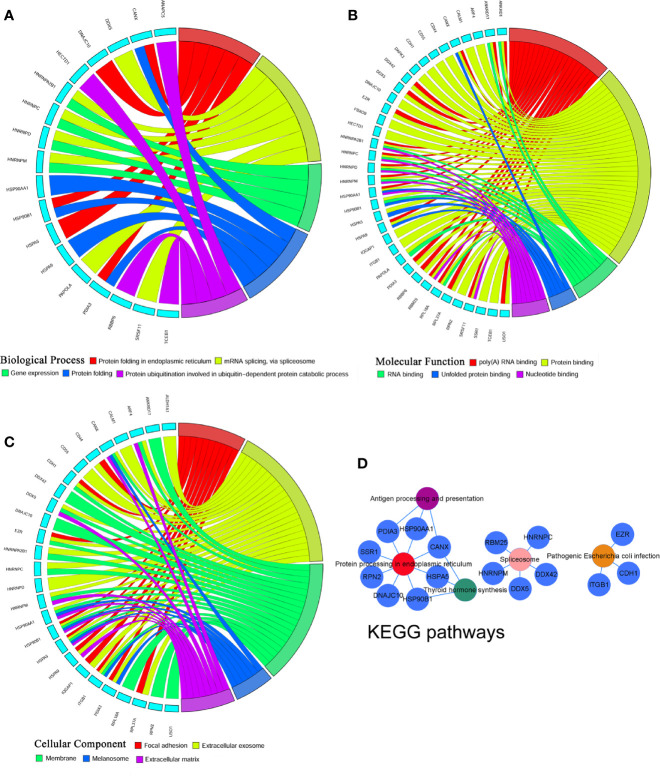
Gene Ontology terms and Kyoto Encyclopedia of Genes and Genomes (KEGG) pathways of the module differentially expressed genes (DEGs) set. **(A)** Biological process, **(B)** Cellular component, **(C)** Molecular function, **(D)** KEGG pathways: Colors from red, pink, yellow, green to purple indicate that the P values are sorted from minimum to maximum.

**Figure 5:**
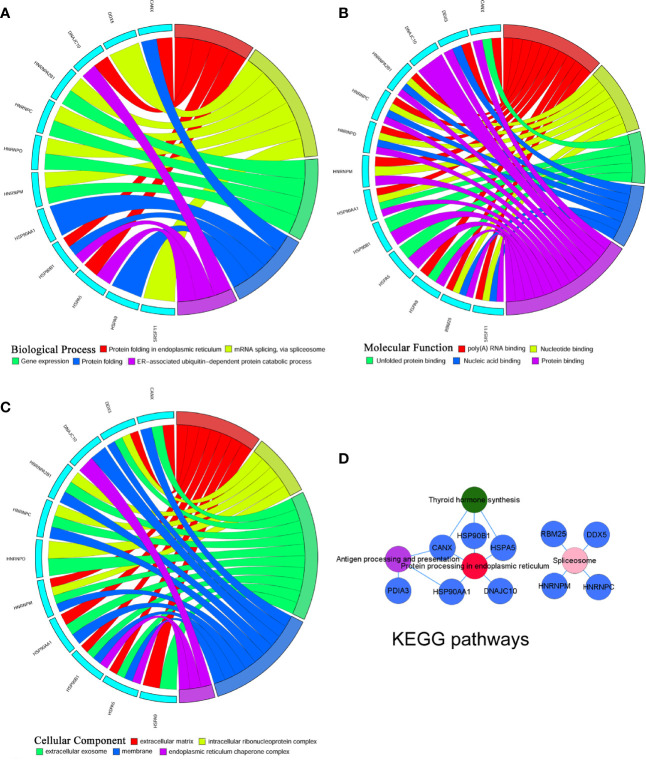
Gene Ontology terms and Kyoto Encyclopedia of Genes and Genomes (KEGG) pathways of the hub differentially expressed genes (DEGs) set. **(A)** Biological process, **(B)** Cellular component, **(C)** Molecular function, **(D)** KEGG pathways: Colors from red, pink, green to purple indicate that the P values are sorted from minimum to maximum.

To identify the most significant DEGs “hub genes” associated with GO lacrimal enlargement, we performed a PPI network construction of all the overlapping DEGs on the basis of the STRING database. Finally, 157 nodes and 282 edges were present in the PPI network complex (**Figure 3A**), in which 16 central node genes were selected with the cut-off criteria of connectivity degree ≥ 10 (**Figure 3B**). Subsequently, module analysis was performed by the plug-in MCODE in Cytoscape software in order to identify the significant modules from the PPI network complex. Then, 6 modules successfully emerged from the above PPI network complex, including 40 genes. According to the selection criteria of MCODE score > 3 and node number > 4, module a, module b, module c and module d were regarded as significant modules (**Figure 3C**). Among them, module A with the highest MCODE score and the maximum node number was considered as the most important module. Therefore, the common genes present in 16 central nodes and module A were selected as hub genes, including *HSP90AA1*, *DNAJC10*, *HNRNPM*, *HNRNPD*, *HNRNPC*, *CANX*, *HSPA5*, *HSP90B1*, *SRSF11*, *DDX5*, *RBM25*, *HNRNPA2B1*, and *HSPA9* (**Figure 3C**, **Table 1**). Besides, function and pathway analyses of DEGs in the modules or hub genes were further carried out. The functional annotations of the genes involved in the modules were protein folding in ER, poly (A) RNA binding, focal adhesion (**Figures 4A–C**). Pathway analysis showed that the module genes mainly had a close relationship with protein processing in ER, spliceosome and pathogenic Escherichia coli infection (**Figure 4D**). Moreover, the 13 hub genes were significantly enriched in multiple functions such as protein folding in ER, poly (A) RNA binding and extracellular matrix (**Figures 5A–C**). And KEGG revealed that these hub genes were enriched in several pathways, especially protein processing in ER (**Figure 5D**).

**Table 1:** The hub genes associated with lacrimal gland enlargement in Graves’ ophthalmopathy.

Gene symbol	GSE105149			GSE58331			Regulation
	False discoveryrate (FDR)	P value	Log (fold change)	False discoveryrate (FDR)	P value	Log (fold change)	
CANX	5.56E-02	2.59E-04	−1.02	3.25E-02	2.35E-03	−1.00	down
HSPA9	4.77E-02	1.22E-04	−1.07	3.24E-02	2.33E-03	−1.35	down
RBM25	1.11E-01	5.34E-03	−1.08	2.42E-02	8.83E-04	−1.58	down
HNRNPC	7.67E-02	1.53E-03	−1.11	3.46E-02	2.78E-03	−1.18	down
DNAJC10	5.80E-02	3.59E-04	−1.13	3.09E-02	2.03E-03	−1.57	down
DDX5	6.31E-02	5.59E-04	−1.14	4.36E-02	5.04E-03	−1.78	down
HNRNPM	3.95E-02	9.20E-06	−1.16	1.77E-02	1.57E-04	−1.45	down
HNRNPD	7.82E-02	1.65E-03	−1.26	3.61E-02	3.14E-03	−1.34	down
HSP90AA1	7.42E-02	1.11E-03	−1.27	2.51E-02	1.01E-03	−1.53	down
HSP90B1	5.43E-02	2.25E-04	−1.27	2.90E-02	1.68E-03	−1.16	down
HSPA5	7.43E-02	1.21E-03	−1.43	3.22E-02	2.30E-03	−1.34	down
HNRNPA2B1	5.01E-02	1.73E-04	−1.43	6.57E-02	1.17E-02	−1.29	down
SRSF11	6.10E-02	4.20E-04	−1.54	2.47E-02	9.66E-04	−1.42	down

According to Gene Ontology and KEGG pathway analyses for the entire DEGs set, 40 module genes and 13 hub genes, we observed that protein processing in ER was the most significant pathway, in which five hub genes including *HSP90AA1*, *HSP90B1*, *DNAJC10*, *HSPA5* and *CANX* were involved.

### Differences in Pathway Activities between GO and Normal Lacrimal Gland

**Figure 6:**
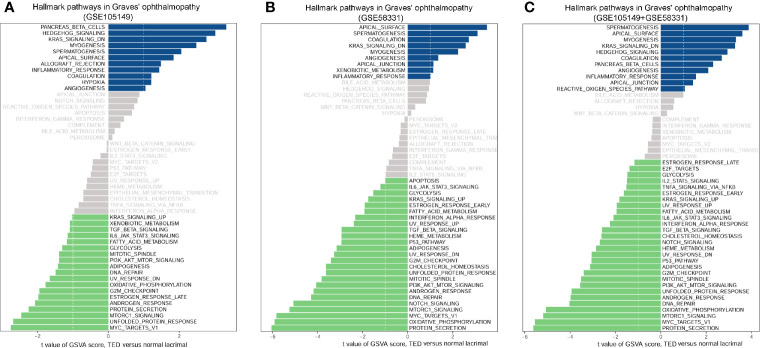
Scores of gene set variation analysis (GSVA) for pathway activities between thyroid eye disease (TED) and normal lacrimal gland. **(A)** GSVA analysis of four TED versus seven normal lacrimal samples of GSE105149. **(B)** GSVA analysis of seven TED versus seven normal lacrimal samples of GSE58331. **(C)** The common GSVA analysis of 11 TED versus 14 normal lacrimal samples of GSE105149 and GSE58331. Shown are t values from a linear model, corrected for patient of origin. DN, down; v1, version.

KEGG analysis requires relatively arbitrary selection of DEGs as input, only a small number of pathways were enriched in GO. In order to correct this selection to some extent, we applied GSVA analysis to investigate the critical pathways of GO. Since GSVA transforms data from a gene to a gene set based on the sample matrix in the coordinate frame, the enrichment pathway of each sample can be evaluated. As a result, GSVA analysis of TED versus normal lacrimal samples significantly revealed four up-regulated and five down-regulated signatures in the TED lacrimal samples of GSE105149 (**Figure 6A**), five up-regulated and 19 down-regulated signatures in the TED lacrimal samples of GSE58331 (**Figure 6B**). The common enriched signals in both GSE105149 and GSE58331 were KRAS_SIGNALING_DN, MYOGENESIS, MYC_TARGETS_V1, UNFOLDED_PROTEIN_RESPONSE, MTORC1_SIGNALING, PROTEIN_SECRETION and ANDROGEN_RESPONSE. In addition, the GSVA analysis of the combined data of GSE105149 and GSE58331 further supported the above results (**Figure 6C**). These results further supported the differences in pathway activities between GO and normal lacrimal gland, and provided novel or related pathways compared with KEGG pathway analysis.

## Discussion

Currently, our understanding of GO pathogenesis is mainly focused on the extraocular muscle and orbital adipose tissue. The molecular origins of lacrimal gland enlargement in GO has not yet been elucidated. In this work, two gene expression profiles from different cohort studies were analyzed to identify DEGs with common changes between lacrimal gland from patients with GO and that from normal groups. Further bioinformatics analysis was performed to investigate the significance of these DEGs, including Gene ontology analysis, KEGG pathway analysis, PPI network construction, hub gene identification and GSVA analysis.

We identified a total of 173 common DEGs which may be involved in the most significant function and pathway in ER, including BPs “Protein folding in ER” “ER to Golgi vesicle-mediated transport”, MFs “Peptidyl-prolyl cis-trans isomerase activity” “Cis-trans isomerase activity” and CCs “Golgi apparatus” “ER” and pathways “Protein processing in ER” “Phagosome”. As we known, ER is like a special factory for eukaryotic cells, in which at least one-third of molecules are synthesized, folded and transported (23). It maintains proper physiological proteostasis that the correctly folded molecules are modified and transported into the Golgi complexes, while the misfolded or unfolded molecules are trapped in the ER together with the molecular chaperone complex. When a large number of misfolded and unfolded molecules are accumulated, ER stress occurs and stimulates the unfolded protein response (UPR). Significantly, UPR intersects with different networks of inflammation, stress signals, and oxidative stress, in which NF-kB and JNK-AP1 pathways are involved (24). Inflammation, as an important pathological change of GO, directly affects the severity and activity of GO. Clinically, glucocorticoids can effectively alleviate the condition of some active moderate to severe GO patients by controlling the inflammatory response (25). In addition, previous reports have confirmed the dysfunctions of NF-kB pathway and oxidative stress in the progression of GO (26–29). In this study, GSVA analysis also showed the dysregulation of protein secretion, oxidative phosphorylation, UPR and inflammatory response in lacrimal samples of GO. Therefore, these above evidences supported the ER and related signaling networks as potential mechanisms of GO lacrimal enlargement.

Protein processing in ER was identified as the most significant pathway associated with the pathogenesis of GO lacrimal enlargement, in which 5 hub genes including *HSP90AA1*, *HSP90B1*, *DNAJC10*, *HSPA5*, and *CANX* were involved. These proteins respectively encoding by *HSP90B1*, *DNAJC10*, *HSPA5*, and *CANX* are all located in the ER. Of them, HSP90AA1 has the highest connectivity in the PPI construction of DEGs, which encodes the well-known cytoplasmic Hsp90α, a highly conserved molecular chaperone that facilitates folding, maturation, structural maintenance and appropriate modification of target molecules (known as clients) (30). These clients are enriched in vesicle-mediated transport, telomere maintenance, mitotic signal transduction, apoptosis, cell-cycle progression, innate immunity and targeted protein degradation (31). For example, the binding of Hsp90α to lipopolysaccharide (LPS) can mediate LPS-induced inflammation, including the secretion of TNF by monocytes (32). Interestingly, LPS-induced inflammation has been proved to affect the differentiation of orbital pre-adipocytes in GO (33). HSP90B1 encodes another Hsp90 isoform GRP94, an ER-resident molecular chaperone, which plays a vital role in the processing and transportation of secreting proteins (34). GRP94 shows many biochemical characteristics with other HSP90 family members, but also exhibits different activities, such as calcium binding, which is required for conditions in the ER (35). Studies have shown that GRP94 functions as a key molecular chaperone for various Toll-like receptors and integrins (36, 37), and also regulates innate and adaptive immunity (38). The absence of GRP94 in the hematopoietic system of mice leads to thrombocytopenia, prolonged bleeding, and massive platelets, which are clinically difficult to distinguish from human Bernard-soulier Syndrome (39). In addition, DNACJ10 and HSPA5 respectively encode the ER-resident proteins ERdj5 and BiP, which are crucial parts of ER-associated degradation (ERAD) complex involved in recognizing and degrading misfolded or unfolded molecules (40). ERAD is an essential quality-control system for ER molecules, in which the correctly folded molecules are transported to the secretion pathway and finally misfolded or unfolded molecules are translocated into the cytosol for proteasome degradation (41). Ushioda et al. confirmed that ERdj5 acts as a reductase that breaks the disulfide bonds of misfolded or unfolded molecules and facilitates ERAD by the physical and functional connections with BiP (42). Furthermore, studies suggested ERdj5 and BiP as the master regulator of calcium homeostasis and redox homeostasis in the ER (43). Similarly, Calnexin (CANX) is a key ER-resident chaperone to help folding and quality control, thus ensuring that only correctly folded and assembled molecules can further enter into the secretion pathway (44). All these findings further deepen our understanding of the important roles of *HSP90AA1*, *HSP90B1*, *DNAJC10*, *HSPA5* and *CANX* in protein processing in ER. Meanwhile, the observation that the down-regulated expression of these 5 hub genes in GO lacrimal samples, further indicated that ER dysfunction may contribute to the enlargement of GO lacrimal gland, and may extend to other pathological manifestations of GO.

In summary, our study presented a comprehensive bioinformatics analysis to identify potential key genes and pathways that may help to uncover the molecular mechanisms of the lacrimal gland enlargement in GO. Our findings firstly revealed that down-regulated expression of HSP90AA1, HSP90B1, DNAJC10, HSPA5, and CANX might generate the dysfunction of protein processing in ER, providing a novel explanation for the etiology and molecular events of the lacrimal enlargement in GO. When further molecular experiments confirmed the findings of the identified candidate genes and pathways in GO, the underlying molecular mechanisms of lacrimal gland enlargement in GO would be elucidated. All of these would contribute to a novel insight into GO pathogenesis.

## Data Availability Statement

The data sets (GSE105149 and GSE58331) for this study can be found in Gene Expression Omnibus (http://www.ncbi.nlm.nih.gov/geo/).

## Author Contributions

WT and YS conceived the experiments. JY, ZM, and XJ analyzed the data. WT drafted the manuscript. All authors contributed to the article and approved the submitted version.

## Funding

This work was supported by Scientific Research Fund of Chengdu Medical College, Grant/Award Number: CYZ18-24/CYZ18-12.

## Conflict of Interest

Author Jia Yao was employed by company Chengdu SuAn Technology Co., Ltd.

The remaining authors declare that the research was conducted in the absence of any commercial or financial relationships that could be construed as a potential conflict of interest.

## References

[1] Wang Y Smith T Current concepts in the molecular pathogenesis of thyroid-associated ophthalmopathy. Invest Ophthalmol Vis Sci (2014) 55(3):1735–48. 10.1167/iovs.14-14002 PMC3968932 24651704

[2] Bartalena L Baldeschi L Boboridis K Eckstein A Kahaly GJ Marcocci C The 2016 European Thyroid Association/European Group on Graves’ Orbitopathy Guidelines for the Management of Graves’ Orbitopathy. Eur Thyroid J (2016) 5(1):9–26. 10.1159/000443828 27099835 PMC4836120

[3] Iyer S Bahn R Immunopathogenesis of Graves’ ophthalmopathy: the role of the TSH receptor. Best Pract Res Clin Endocrinol Metab (2012) 26(3):281–9. 10.1016/j.beem.2011.10.003 PMC3361679 22632365

[4] Bahn RS Graves’ ophthalmopathy. N Engl J Med (2010) 362(8):726–38. 10.1056/NEJMra0905750 PMC3902010 20181974

[5] Bahn RS Current Insights into the Pathogenesis of Graves’ Ophthalmopathy. Horm Metab Res (2015) 47(10):773–8. 10.1055/s-0035-1555762 26361262

[6] Smith T J Hegedus L Graves’ Disease. N Engl J Med (2016) 375(16):1552–65. 10.1056/NEJMra1510030 27797318

[7] Wescombe L Lahooti H Gopinath B Wall JR The cardiac calsequestrin gene (CASQ2) is up-regulated in the thyroid in patients with Graves’ ophthalmopathy–support for a role of autoimmunity against calsequestrin as the triggering event. Clin Endocrinol (Oxf) (2010) 73(4):522–8. 10.1111/j.1365-2265.2009.03753.x 20039900

[8] Kumar S Leontovich A Coenen MJ Bahn RS Gene expression profiling of orbital adipose tissue from patients with Graves’ ophthalmopathy: a potential role for secreted frizzled-related protein-1 in orbital adipogenesis. J Clin Endocrinol Metab (2005) 90(8):4730–5. 10.1210/jc.2004-2239 PMC1236982 15886250

[9] Lantz M Vondrichova T Parikh H Frenander C Ridderstrale M Asman P Overexpression of immediate early genes in active Graves’ ophthalmopathy. J Clin Endocrinol Metab (2005) 90(8):4784–91. 10.1210/jc.2004-2275 15928252

[10] Ezra DG Krell J Rose GE Bailly M Stebbing J Castellano L Transcriptome-level microarray expression profiling implicates IGF-1 and Wnt signalling dysregulation in the pathogenesis of thyroid-associated orbitopathy. J Clin Pathol (2012) 65(7):608–13. 10.1136/jclinpath-2012-200719 PMC7611799 22554965

[11] Khong JJ Wang LY Smyth GK McNab AA Hardy TG Selva D Differential Gene Expression Profiling of Orbital Adipose Tissue in Thyroid Orbitopathy. Invest Ophthalmol Vis Sci (2015) 56(11):6438–47. 10.1167/iovs.15-17185 26444724

[12] Mou P Chen Z Jiang L Cheng J Wei R PTX3: A Potential Biomarker in Thyroid Associated Ophthalmopathy. BioMed Res Int (2018) 2018):5961974. 10.1155/2018/5961974 29675428 PMC5838458

[13] Tao W Ayala-Haedo JA Field MG Pelaez D Wester ST RNA-Sequencing Gene Expression Profiling of Orbital Adipose-Derived Stem Cell Population Implicate HOX Genes and WNT Signaling Dysregulation in the Pathogenesis of Thyroid-Associated Orbitopathy. Invest Ophthalmol Vis Sci (2017) 58(14):6146–58. 10.1167/iovs.17-22237 PMC5718600 29214313

[14] Lee BW Kumar VB Biswas P Ko AC Alameddine RM Granet DB Transcriptome Analysis of Orbital Adipose Tissue in Active Thyroid Eye Disease Using Next Generation RNA Sequencing Technology. Open Ophthalmol J (2018) 12:41–52. 10.2174/1874364101812010041 29760827 PMC5906971

[15] Zhang L Masetti G Colucci G Salvi M Covelli D Eckstein A Combining micro-RNA and protein sequencing to detect robust biomarkers for Graves’ disease and orbitopathy. Sci Rep (2018) 8(1):8386. 10.1038/s41598-018-26700-1 29849043 PMC5976672

[16] Rosenbaum JT Choi D Harrington CA Wilson DJ Grossniklaus HE Sibley CH Gene Expression Profiling and Heterogeneity of Nonspecific Orbital Inflammation Affecting the Lacrimal Gland. JAMA Ophthalmol (2017) 135(11):1156–62. 10.1001/jamaophthalmol.2017.3458 PMC6033262 28975236

[17] Wong AJ Planck SR Choi D Harrington CA Troxell ML Houghton DC IgG4 immunostaining and its implications in orbital inflammatory disease. PloS One (2014) 9(10):e109847. 10.1371/journal.pone.0109847 25303270 PMC4193851

[18] Rosenbaum JT Choi D Wong A Wilson DJ Grossniklaus HE Harrington CA The Role of the Immune Response in the Pathogenesis of Thyroid Eye Disease: A Reassessment. PloS One (2015) 10(9):e0137654. 10.1371/journal.pone.0137654 26371757 PMC4570801

[19] Rosenbaum JT Choi D Wilson DJ Grossniklaus HE Sibley CH Harrington CA Molecular diagnosis of orbital inflammatory disease. Exp Mol Pathol (2015) 98(2):225–9. 10.1016/j.yexmp.2015.01.009 PMC4388776 25595914

[20] Rosenbaum JT Choi D Wilson DJ Grossniklaus HE Harrington CA Sibley CH Parallel Gene Expression Changes in Sarcoidosis Involving the Lacrimal Gland, Orbital Tissue, or Blood. JAMA Ophthalmol (2015) 133(7):770–7. 10.1001/jamaophthalmol.2015.0726 PMC5021543 25880323

[21] Rosenbaum JT Choi D Wilson DJ Grossniklaus HE Harrington CA Dailey RA Fibrosis, gene expression and orbital inflammatory disease. Br J Ophthalmol (2015) 99(10):1424–9. 10.1136/bjophthalmol-2015-306614 PMC4912840 26038391

[22] Hanzelmann S Castelo R Guinney J GSVA: gene set variation analysis for microarray and RNA-seq data. BMC Bioinf (2013) 14:7. 10.1186/1471-2105-14-7 PMC3618321 23323831

[23] Wang M Kaufman RJ Protein misfolding in the endoplasmic reticulum as a conduit to human disease. Nature (2016) 529(7586):326–35. 10.1038/nature17041 26791723

[24] Hotamisligil GS Endoplasmic reticulum stress and the inflammatory basis of metabolic disease. Cell (2010) 140(6):900–17. 10.1016/j.cell.2010.02.034 PMC2887297 20303879

[25] Stan MN Garrity JA Bahn RS The evaluation and treatment of graves ophthalmopathy. Med Clin North Am (2012) 96(2):311–28. 10.1016/j.mcna.2012.01.014 PMC3898790 22443978

[26] Luo LH Li DM Wang YL Wang K Gao LX Li S Tim3/galectin-9 alleviates the inflammation of TAO patients via suppressing Akt/NF-kB signaling pathway. Biochem Biophys Res Commun (2017) 491(4):966–72. 10.1016/j.bbrc.2017.07.144 28756232

[27] Li H Yuan Y Zhang Y He Q Xu R Ge F Celastrol inhibits IL-1beta-induced inflammation in orbital fibroblasts through the suppression of NF-kappaB activity. Mol Med Rep (2016) 14(3):2799–806. 10.3892/mmr.2016.5570 27484716

[28] Choi W Li Y Ji YS Yoon KC Oxidative stress markers in tears of patients with Graves’ orbitopathy and their correlation with clinical activity score. BMC Ophthalmol (2018) 18(1):303. 10.1186/s12886-018-0969-x 30463536 PMC6249971

[29] Kau HC Wu SB Tsai CC Liu CJ Wei YH Cigarette Smoke Extract-Induced Oxidative Stress and Fibrosis-Related Genes Expression in Orbital Fibroblasts from Patients with Graves’ Ophthalmopathy. Oxid Med Cell Longev (2016) 2016:4676289. 10.1155/2016/4676289 27340508 PMC4909929

[30] Taipale M Jarosz DF Lindquist S HSP90 at the hub of protein homeostasis: emerging mechanistic insights. Nat Rev Mol Cell Biol (2010) 11(7):515–28. 10.1038/nrm2918 20531426

[31] Hartl FU Bracher A Hayer-Hartl M Molecular chaperones in protein folding and proteostasis. Nature (2011) 475(7356):324–32. 10.1038/nature10317 21776078

[32] Triantafilou K Triantafilou M Dedrick RL A CD14-independent LPS receptor cluster. Nat Immunol (2001) 2(4):338–45. 10.1038/86342 11276205

[33] Yi WS Xu XL Effects of LPS-induced inflammation on differentiation of orbital pre-adipocytes in thyroid-associated ophthalmopathy. Zhonghua Yan Ke Za Zhi (2011) 47(2):156–61. 21426847

[34] Huck JD Que NL Hong F Li Z Gewirth DT Structural and Functional Analysis of GRP94 in the Closed State Reveals an Essential Role for the Pre-N Domain and a Potential Client-Binding Site. Cell Rep (2017) 20(12):2800–9. 10.1016/j.celrep.2017.08.079 PMC5608278 28930677

[35] Marzec M Eletto D Argon Y GRP94: An HSP90-like protein specialized for protein folding and quality control in the endoplasmic reticulum. Biochim Biophys Acta (2012) 1823(3):774–87. 10.1016/j.bbamcr.2011.10.013 PMC3443595 22079671

[36] Yang Y Liu B Dai J Srivastava PK Zammit DJ Lefrancois L Heat shock protein gp96 is a master chaperone for toll-like receptors and is important in the innate function of macrophages. Immunity (2007) 26(2):215–26. 10.1016/j.immuni.2006.12.005 PMC2847270 17275357

[37] Staron M Yang Y Liu B Li J Shen Y Zuniga-Pflucker JC gp96, an endoplasmic reticulum master chaperone for integrins and Toll-like receptors, selectively regulates early T and B lymphopoiesis. Blood (2010) 115(12):2380–90. 10.1182/blood-2009-07-233031 PMC2845896 19965672

[38] Randow F Seed B Endoplasmic reticulum chaperone gp96 is required for innate immunity but not cell viability. Nat Cell Biol (2001) 3(10):891–6. 10.1038/ncb1001-891 11584270

[39] Staron M Wu S Hong F Stojanovic A Du X Bona R Heat-shock protein gp96/grp94 is an essential chaperone for the platelet glycoprotein Ib-IX-V complex. Blood (2011) 117(26):7136–44. 10.1182/blood-2011-01-330464 PMC3143555 21576699

[40] Hagiwara M Maegawa K Suzuki M Ushioda R Araki K Matsumoto Y Structural basis of an ERAD pathway mediated by the ER-resident protein disulfide reductase ERdj5. Mol Cell (2011) 41(4):432–44. 10.1016/j.molcel.2011.01.021 21329881

[41] Ellgaard L Helenius A Quality control in the endoplasmic reticulum. Nat Rev Mol Cell Biol (2003) 4(3):181–91. 10.1038/nrm1052 12612637

[42] Ushioda R Hoseki J Araki K Jansen G Thomas DY Nagata K ERdj5 is required as a disulfide reductase for degradation of misfolded proteins in the ER. Science (2008) 321(5888):569–72. 10.1126/science.1159293 18653895

[43] Ushioda R Miyamoto A Inoue M Watanabe S Okumura M Maegawa K II Redox-assisted regulation of Ca2+ homeostasis in the endoplasmic reticulum by disulfide reductase ERdj5. Proc Natl Acad Sci U.S.A. (2016) 113(41):E6055–63. 10.1073/pnas.1605818113 PMC5068290 27694578

[44] Schrag JD Bergeron JJ Li Y Borisova S Hahn M Thomas DY The Structure of calnexin, an ER chaperone involved in quality control of protein folding. Mol Cell (2001) 8(3):633–44. 10.1016/s1097-2765(01)00318-5 11583625

