# Supplementary material for: Microarray Data of Lacrimal Gland Implicates Dysregulated Protein Processing in Endoplasmic Reticulum in Graves’ Ophthalmopathy
Source: Front Endocrinol (Lausanne). 2021 Feb 3;11:571151. doi: 10.3389/fendo.2020.571151 (PMC7888479; doi:10.3389/fendo.2020.571151)
Supplement: Supplementary file 1 [file Table_1.docx]

**Table S1. The common differentially expressed genes in GSE105149 and GSE58331**

| **Gene symbol** | **GSE105149** | | |  | **GSE58331** | | | **Regulation** |
| --- | --- | --- | --- | --- | --- | --- | --- | --- |
|  | **FDR** | **P value** | **Log (fold change)** |  | **FDR** | **P value** | **Log (fold change)** |  |
| HLA-DQA1 | 1.35E-01 | 9.57E-03 | 3.32 |  | 4.23E-02 | 4.63E-03 | 2.87 | up |
| RPL18A | 4.57E-02 | 6.36E-05 | 2.16 |  | 4.40E-02 | 5.13E-03 | 1.12 | up |
| SNHG17 | 8.85E-02 | 2.51E-03 | 2.00 |  | 6.45E-02 | 1.13E-02 | 1.24 | up |
| SNORA74A | 4.05E-02 | 3.06E-05 | 1.97 |  | 6.02E-02 | 9.97E-03 | 1.23 | up |
| SNORA71B | 3.95E-02 | 6.50E-06 | 1.86 |  | 6.11E-02 | 1.03E-02 | 1.36 | up |
| ASCL2 | 5.43E-02 | 2.14E-04 | 1.82 |  | 3.36E-02 | 2.59E-03 | 1.46 | up |
| POM121L10P | 6.62E-02 | 6.89E-04 | 1.28 |  | 2.21E-02 | 6.62E-04 | 1.37 | up |
| POM121L1P | 6.62E-02 | 6.89E-04 | 1.28 |  | 2.21E-02 | 6.62E-04 | 1.37 | up |
| POM121L4P | 6.62E-02 | 6.89E-04 | 1.28 |  | 2.21E-02 | 6.62E-04 | 1.37 | up |
| POM121L8P | 6.62E-02 | 6.89E-04 | 1.28 |  | 2.21E-02 | 6.62E-04 | 1.37 | up |
| POM121L9P | 6.62E-02 | 6.89E-04 | 1.28 |  | 2.21E-02 | 6.62E-04 | 1.37 | up |
| SNORA50C | 9.34E-02 | 3.01E-03 | 1.22 |  | 2.04E-02 | 4.70E-04 | 1.41 | up |
| PTMS | 5.74E-02 | 3.44E-04 | 1.18 |  | 5.10E-02 | 7.18E-03 | 1.04 | up |
| PDE8A | 4.77E-02 | 1.14E-04 | 1.14 |  | 2.05E-02 | 4.82E-04 | 1.65 | up |
| MUC3A | 2.23E-01 | 3.82E-02 | 1.12 |  | 2.66E-02 | 1.22E-03 | 1.13 | up |
| AP1S3 | 6.78E-02 | 7.58E-04 | 1.10 |  | 2.12E-02 | 5.50E-04 | 1.15 | up |
| DAPK3 | 8.08E-02 | 1.87E-03 | 1.10 |  | 2.27E-02 | 7.18E-04 | 1.35 | up |
| AGBL4 | 5.43E-02 | 2.22E-04 | 1.07 |  | 4.59E-02 | 5.66E-03 | 1.01 | up |
| ZNF414 | 8.67E-02 | 2.31E-03 | 1.06 |  | 2.08E-02 | 5.19E-04 | 1.06 | up |
| LIM2 | 5.43E-02 | 2.16E-04 | 1.01 |  | 3.53E-02 | 2.95E-03 | 1.32 | up |
| TPM4 | 7.31E-02 | 1.06E-03 | -1.00 |  | 6.67E-02 | 1.21E-02 | -1.04 | down |
| TM9SF2 | 8.69E-02 | 2.33E-03 | -1.00 |  | 1.97E-02 | 4.12E-04 | -1.37 | down |
| ARL1 | 7.80E-02 | 1.61E-03 | -1.00 |  | 1.76E-02 | 1.38E-04 | -1.37 | down |
| FOLH1B | 9.67E-02 | 3.30E-03 | -1.00 |  | 8.16E-02 | 1.75E-02 | -1.72 | down |
| DEGS1 | 6.57E-02 | 6.74E-04 | -1.00 |  | 1.72E-02 | 1.01E-04 | -1.13 | down |
| ARL17A | 1.93E-01 | 2.66E-02 | -1.01 |  | 1.41E-01 | 4.53E-02 | -1.00 | down |
| ARL17B | 1.93E-01 | 2.66E-02 | -1.01 |  | 1.41E-01 | 4.53E-02 | -1.00 | down |
| RPN2 | 9.07E-02 | 2.72E-03 | -1.01 |  | 1.72E-02 | 1.04E-04 | -1.26 | down |
| C1QBP | 3.95E-02 | 9.70E-06 | -1.01 |  | 3.10E-02 | 2.07E-03 | -1.31 | down |
| ZNF644 | 1.43E-01 | 1.17E-02 | -1.02 |  | 3.66E-02 | 3.23E-03 | -1.24 | down |
| SLC30A5 | 7.31E-02 | 1.05E-03 | -1.02 |  | 5.87E-02 | 9.49E-03 | -1.12 | down |
| RBBP6 | 6.57E-02 | 6.73E-04 | -1.02 |  | 1.77E-02 | 1.71E-04 | -1.58 | down |
| MTUS1 | 1.06E-01 | 4.41E-03 | -1.02 |  | 2.47E-02 | 9.55E-04 | -1.09 | down |
| CANX | 5.56E-02 | 2.59E-04 | -1.02 |  | 3.25E-02 | 2.35E-03 | -1.00 | down |
| FEM1B | 7.82E-02 | 1.66E-03 | -1.02 |  | 5.11E-02 | 7.20E-03 | -1.09 | down |
| PPP4R3A | 5.71E-02 | 3.30E-04 | -1.02 |  | 1.90E-02 | 3.38E-04 | -1.38 | down |
| TBC1D30 | 2.45E-01 | 4.70E-02 | -1.02 |  | 7.05E-02 | 1.33E-02 | -1.26 | down |
| PAPOLA | 7.24E-02 | 9.92E-04 | -1.03 |  | 3.08E-02 | 2.02E-03 | -1.39 | down |
| RAB27B | 1.14E-01 | 5.83E-03 | -1.03 |  | 2.95E-02 | 1.79E-03 | -1.71 | down |
| ITGA6 | 1.03E-01 | 4.02E-03 | -1.03 |  | 1.84E-02 | 2.55E-04 | -1.60 | down |
| GPCPD1 | 6.57E-02 | 6.62E-04 | -1.03 |  | 1.88E-02 | 2.84E-04 | -1.32 | down |
| ANAPC5 | 1.28E-01 | 8.02E-03 | -1.03 |  | 1.76E-02 | 1.29E-04 | -1.24 | down |
| SMARCA2 | 4.77E-02 | 1.20E-04 | -1.03 |  | 7.68E-02 | 1.56E-02 | -1.01 | down |
| ZNF75A | 1.01E-01 | 3.77E-03 | -1.03 |  | 6.10E-02 | 1.02E-02 | -1.09 | down |
| MALAT1 | 5.32E-02 | 1.96E-04 | -1.04 |  | 9.14E-02 | 2.16E-02 | -1.00 | down |
| PEX3 | 3.98E-02 | 2.19E-05 | -1.04 |  | 1.78E-02 | 1.76E-04 | -1.34 | down |
| CD9 | 9.33E-02 | 2.97E-03 | -1.04 |  | 2.85E-02 | 1.56E-03 | -1.31 | down |
| TCEB1 | 1.09E-01 | 4.87E-03 | -1.04 |  | 2.21E-02 | 6.52E-04 | -1.16 | down |
| GMDS | 5.46E-02 | 2.36E-04 | -1.04 |  | 8.02E-02 | 1.70E-02 | -1.28 | down |
| IFI16 | 8.44E-02 | 2.09E-03 | -1.05 |  | 3.92E-02 | 3.90E-03 | -1.29 | down |
| CD55 | 2.07E-01 | 3.18E-02 | -1.05 |  | 3.13E-02 | 2.11E-03 | -1.43 | down |
| HEXB | 4.05E-02 | 3.06E-05 | -1.05 |  | 2.44E-02 | 9.32E-04 | -1.13 | down |
| CD44 | 4.96E-02 | 1.62E-04 | -1.05 |  | 3.14E-02 | 2.14E-03 | -1.49 | down |
| YLPM1 | 8.58E-02 | 2.23E-03 | -1.05 |  | 1.72E-02 | 9.70E-05 | -1.31 | down |
| IQGAP1 | 6.66E-02 | 7.15E-04 | -1.05 |  | 2.44E-02 | 9.16E-04 | -1.32 | down |
| ERBB3 | 7.43E-02 | 1.18E-03 | -1.05 |  | 8.85E-02 | 2.03E-02 | -1.39 | down |
| CP | 2.11E-01 | 3.35E-02 | -1.05 |  | 3.22E-02 | 2.30E-03 | -1.90 | down |
| PIK3C2A | 5.59E-02 | 2.62E-04 | -1.05 |  | 3.54E-02 | 2.98E-03 | -1.03 | down |
| CASK | 4.51E-02 | 5.11E-05 | -1.06 |  | 2.58E-02 | 1.09E-03 | -1.26 | down |
| FAM133B | 1.12E-01 | 5.56E-03 | -1.06 |  | 3.97E-02 | 4.04E-03 | -1.03 | down |
| FBXO9 | 4.77E-02 | 1.18E-04 | -1.06 |  | 3.29E-02 | 2.44E-03 | -1.15 | down |
| KIAA1033 | 5.74E-02 | 3.44E-04 | -1.06 |  | 2.71E-02 | 1.32E-03 | -1.17 | down |
| C1orf27 | 1.18E-01 | 6.42E-03 | -1.06 |  | 3.47E-02 | 2.82E-03 | -1.33 | down |
| BOD1L1 | 7.43E-02 | 1.22E-03 | -1.06 |  | 8.16E-02 | 1.75E-02 | -1.18 | down |
| PSMA1 | 4.63E-02 | 7.12E-05 | -1.06 |  | 2.83E-02 | 1.54E-03 | -1.24 | down |
| MIA3 | 6.64E-02 | 7.05E-04 | -1.07 |  | 3.27E-02 | 2.40E-03 | -1.01 | down |
| LARS | 4.79E-02 | 1.35E-04 | -1.07 |  | 3.48E-02 | 2.84E-03 | -1.11 | down |
| HSPA9 | 4.77E-02 | 1.22E-04 | -1.07 |  | 3.24E-02 | 2.33E-03 | -1.35 | down |
| KRR1 | 7.43E-02 | 1.18E-03 | -1.07 |  | 4.24E-02 | 4.68E-03 | -1.47 | down |
| ATXN7 | 7.42E-02 | 1.12E-03 | -1.07 |  | 8.25E-02 | 1.79E-02 | -1.09 | down |
| DDX42 | 4.63E-02 | 7.06E-05 | -1.07 |  | 2.67E-02 | 1.26E-03 | -1.02 | down |
| HSD17B4 | 5.46E-02 | 2.40E-04 | -1.08 |  | 2.21E-02 | 6.57E-04 | -1.14 | down |
| RBM25 | 1.11E-01 | 5.34E-03 | -1.08 |  | 2.42E-02 | 8.83E-04 | -1.58 | down |
| ESYT2 | 4.79E-02 | 1.41E-04 | -1.09 |  | 3.36E-02 | 2.58E-03 | -1.25 | down |
| SEC23B | 1.11E-01 | 5.31E-03 | -1.10 |  | 4.96E-02 | 6.71E-03 | -1.06 | down |
| ATP6V1G2 | 5.66E-02 | 3.09E-04 | -1.10 |  | 4.25E-02 | 4.70E-03 | -1.01 | down |
| DDX39B | 5.66E-02 | 3.09E-04 | -1.10 |  | 4.25E-02 | 4.70E-03 | -1.01 | down |
| TOX4 | 4.77E-02 | 1.11E-04 | -1.10 |  | 2.79E-02 | 1.45E-03 | -1.46 | down |
| DNAJC7 | 5.66E-02 | 3.08E-04 | -1.11 |  | 1.01E-01 | 2.57E-02 | -1.01 | down |
| ALDOA | 1.47E-01 | 1.26E-02 | -1.11 |  | 3.71E-02 | 3.37E-03 | -1.31 | down |
| HNRNPC | 7.67E-02 | 1.53E-03 | -1.11 |  | 3.46E-02 | 2.78E-03 | -1.18 | down |
| TTC3 | 5.74E-02 | 3.52E-04 | -1.12 |  | 3.75E-02 | 3.51E-03 | -1.45 | down |
| HECTD1 | 4.77E-02 | 8.97E-05 | -1.12 |  | 2.11E-02 | 5.38E-04 | -1.20 | down |
| NAA15 | 5.71E-02 | 3.33E-04 | -1.12 |  | 2.76E-02 | 1.38E-03 | -1.30 | down |
| ATRX | 7.30E-02 | 1.05E-03 | -1.12 |  | 2.95E-02 | 1.80E-03 | -1.53 | down |
| SAR1B | 8.08E-02 | 1.84E-03 | -1.12 |  | 2.97E-02 | 1.83E-03 | -1.32 | down |
| FKBP11 | 8.00E-02 | 1.79E-03 | -1.12 |  | 1.78E-02 | 1.78E-04 | -1.23 | down |
| DNAJC10 | 5.80E-02 | 3.59E-04 | -1.13 |  | 3.09E-02 | 2.03E-03 | -1.57 | down |
| STAM2 | 3.98E-02 | 2.17E-05 | -1.13 |  | 1.78E-02 | 1.85E-04 | -1.18 | down |
| PILRB | 6.32E-02 | 5.61E-04 | -1.13 |  | 4.46E-02 | 5.30E-03 | -1.06 | down |
| PPIG | 5.35E-02 | 2.06E-04 | -1.13 |  | 3.36E-02 | 2.59E-03 | -1.14 | down |
| AASDHPPT | 6.57E-02 | 6.71E-04 | -1.13 |  | 1.84E-02 | 2.56E-04 | -1.18 | down |
| ANKHD1 | 4.77E-02 | 8.34E-05 | -1.14 |  | 5.04E-02 | 6.97E-03 | -1.04 | down |
| LRRFIP1 | 4.33E-02 | 4.68E-05 | -1.14 |  | 1.77E-02 | 1.66E-04 | -1.37 | down |
| DDX5 | 6.31E-02 | 5.59E-04 | -1.14 |  | 4.36E-02 | 5.04E-03 | -1.78 | down |
| TFAP2A | 5.46E-02 | 2.30E-04 | -1.14 |  | 1.33E-02 | 4.24E-06 | -1.40 | down |
| SSR1 | 7.10E-02 | 9.32E-04 | -1.14 |  | 1.77E-02 | 1.63E-04 | -1.14 | down |
| FOXP1 | 3.98E-02 | 1.25E-05 | -1.15 |  | 4.22E-02 | 4.62E-03 | -1.03 | down |
| NEAT1 | 4.77E-02 | 1.07E-04 | -1.15 |  | 1.93E-02 | 3.81E-04 | -1.15 | down |
| DPP8 | 6.25E-02 | 5.06E-04 | -1.15 |  | 4.22E-02 | 4.62E-03 | -1.47 | down |
| ARF4 | 1.45E-01 | 1.22E-02 | -1.15 |  | 4.83E-02 | 6.30E-03 | -1.30 | down |
| HNRNPM | 3.95E-02 | 9.20E-06 | -1.16 |  | 1.77E-02 | 1.57E-04 | -1.45 | down |
| UBXN4 | 6.16E-02 | 4.43E-04 | -1.16 |  | 2.21E-02 | 6.51E-04 | -1.13 | down |
| CSNK1A1 | 1.01E-01 | 3.81E-03 | -1.16 |  | 3.22E-02 | 2.30E-03 | -1.26 | down |
| PDIA3 | 4.77E-02 | 1.29E-04 | -1.16 |  | 2.51E-02 | 1.00E-03 | -1.05 | down |
| RBM39 | 1.02E-01 | 3.94E-03 | -1.16 |  | 7.23E-02 | 1.40E-02 | -1.03 | down |
| ZBTB44 | 6.90E-02 | 8.03E-04 | -1.16 |  | 4.37E-02 | 5.05E-03 | -1.30 | down |
| NEMF | 6.25E-02 | 5.00E-04 | -1.17 |  | 3.49E-02 | 2.86E-03 | -1.53 | down |
| ZNF12 | 7.08E-02 | 9.21E-04 | -1.17 |  | 2.71E-02 | 1.32E-03 | -1.25 | down |
| USO1 | 6.14E-02 | 4.36E-04 | -1.17 |  | 5.01E-02 | 6.87E-03 | -1.20 | down |
| WDR1 | 3.98E-02 | 1.82E-05 | -1.17 |  | 2.07E-02 | 5.08E-04 | -1.32 | down |
| NF2 | 6.27E-02 | 5.19E-04 | -1.18 |  | 5.22E-02 | 7.52E-03 | -1.00 | down |
| DPP4 | 1.21E-01 | 6.89E-03 | -1.18 |  | 7.56E-02 | 1.52E-02 | -1.00 | down |
| SON | 6.77E-02 | 7.45E-04 | -1.18 |  | 2.77E-02 | 1.41E-03 | -1.15 | down |
| AHCTF1 | 4.96E-02 | 1.60E-04 | -1.18 |  | 5.70E-02 | 8.89E-03 | -1.01 | down |
| ABI1 | 4.77E-02 | 7.73E-05 | -1.18 |  | 5.03E-02 | 6.93E-03 | -1.21 | down |
| ATP6V1A | 4.05E-02 | 2.80E-05 | -1.19 |  | 3.79E-02 | 3.60E-03 | -1.33 | down |
| SLC5A1 | 5.89E-02 | 3.90E-04 | -1.19 |  | 6.53E-02 | 1.16E-02 | -1.39 | down |
| AGAP6 | 4.88E-02 | 1.51E-04 | -1.20 |  | 6.85E-02 | 1.27E-02 | -1.00 | down |
| SYNCRIP | 5.61E-02 | 2.86E-04 | -1.20 |  | 1.94E-02 | 3.87E-04 | -1.43 | down |
| PAXBP1 | 8.80E-02 | 2.47E-03 | -1.20 |  | 4.82E-02 | 6.28E-03 | -1.09 | down |
| GLG1 | 4.77E-02 | 7.97E-05 | -1.21 |  | 3.08E-02 | 2.02E-03 | -1.09 | down |
| ITGB1 | 5.43E-02 | 2.27E-04 | -1.21 |  | 5.57E-02 | 8.50E-03 | -1.15 | down |
| PTP4A1 | 2.11E-01 | 3.36E-02 | -1.21 |  | 3.41E-02 | 2.67E-03 | -1.78 | down |
| KPNA1 | 6.38E-02 | 5.87E-04 | -1.21 |  | 3.09E-02 | 2.05E-03 | -1.31 | down |
| CDH1 | 5.61E-02 | 2.77E-04 | -1.21 |  | 4.03E-02 | 4.20E-03 | -1.51 | down |
| ZNF207 | 6.18E-02 | 4.51E-04 | -1.22 |  | 6.16E-02 | 1.04E-02 | -1.35 | down |
| ANGEL2 | 1.12E-01 | 5.51E-03 | -1.22 |  | 8.70E-02 | 1.97E-02 | -1.05 | down |
| ZNF638 | 7.75E-02 | 1.59E-03 | -1.23 |  | 2.16E-02 | 5.95E-04 | -1.03 | down |
| EZR | 5.66E-02 | 3.13E-04 | -1.24 |  | 3.86E-02 | 3.75E-03 | -1.45 | down |
| RSRP1 | 5.88E-02 | 3.84E-04 | -1.24 |  | 3.72E-02 | 3.43E-03 | -1.29 | down |
| NUMB | 7.66E-02 | 1.48E-03 | -1.24 |  | 4.24E-02 | 4.68E-03 | -1.08 | down |
| ANKRD36B | 9.25E-02 | 2.90E-03 | -1.25 |  | 5.76E-02 | 9.11E-03 | -1.48 | down |
| TECPR1 | 1.77E-01 | 2.10E-02 | -1.25 |  | 5.34E-02 | 7.87E-03 | -1.29 | down |
| ZCCHC2 | 8.72E-02 | 2.36E-03 | -1.25 |  | 2.42E-02 | 8.87E-04 | -1.33 | down |
| GUCY1A2 | 1.46E-01 | 1.23E-02 | -1.26 |  | 1.88E-02 | 2.86E-04 | -1.05 | down |
| HNRNPD | 7.82E-02 | 1.65E-03 | -1.26 |  | 3.61E-02 | 3.14E-03 | -1.34 | down |
| PLA2R1 | 9.07E-02 | 2.71E-03 | -1.27 |  | 1.27E-01 | 3.83E-02 | -1.53 | down |
| ANKRD17 | 3.95E-02 | 9.52E-06 | -1.27 |  | 3.42E-02 | 2.69E-03 | -1.09 | down |
| HSP90AA1 | 7.42E-02 | 1.11E-03 | -1.27 |  | 2.51E-02 | 1.01E-03 | -1.53 | down |
| RAB1A | 5.97E-02 | 4.04E-04 | -1.27 |  | 3.90E-02 | 3.85E-03 | -1.54 | down |
| BCLAF1 | 4.79E-02 | 1.39E-04 | -1.27 |  | 1.77E-02 | 1.56E-04 | -1.60 | down |
| HSP90B1 | 5.43E-02 | 2.25E-04 | -1.27 |  | 2.90E-02 | 1.68E-03 | -1.16 | down |
| YWHAB | 4.63E-02 | 6.86E-05 | -1.29 |  | 3.60E-02 | 3.11E-03 | -1.35 | down |
| SORBS2 | 8.48E-02 | 2.13E-03 | -1.30 |  | 6.00E-02 | 9.91E-03 | -1.27 | down |
| SELT | 7.06E-02 | 9.08E-04 | -1.30 |  | 2.01E-02 | 4.46E-04 | -1.44 | down |
| ZRANB2 | 7.59E-02 | 1.33E-03 | -1.30 |  | 7.99E-02 | 1.68E-02 | -1.16 | down |
| MCM3AP | 1.02E-01 | 3.89E-03 | -1.31 |  | 3.03E-02 | 1.95E-03 | -1.15 | down |
| PSMA3 | 5.80E-02 | 3.68E-04 | -1.32 |  | 4.13E-02 | 4.42E-03 | -1.10 | down |
| FOLH1 | 5.61E-02 | 2.73E-04 | -1.32 |  | 9.31E-02 | 2.23E-02 | -1.06 | down |
| SRPRA | 7.66E-02 | 1.47E-03 | -1.32 |  | 3.36E-02 | 2.58E-03 | -1.01 | down |
| CALU | 4.77E-02 | 1.17E-04 | -1.34 |  | 3.75E-02 | 3.51E-03 | -1.06 | down |
| GALNT7 | 7.59E-02 | 1.33E-03 | -1.34 |  | 1.93E-02 | 3.73E-04 | -1.92 | down |
| ALDH1A1 | 6.25E-02 | 4.81E-04 | -1.35 |  | 2.12E-02 | 5.59E-04 | -1.17 | down |
| CALM1 | 5.43E-02 | 2.17E-04 | -1.36 |  | 1.64E-02 | 7.09E-05 | -1.63 | down |
| GOLGA8B | 5.80E-02 | 3.60E-04 | -1.37 |  | 1.37E-02 | 2.44E-05 | -2.11 | down |
| PDLIM5 | 5.43E-02 | 2.13E-04 | -1.37 |  | 2.41E-02 | 8.51E-04 | -1.22 | down |
| POLR2J4 | 4.34E-02 | 4.77E-05 | -1.38 |  | 5.36E-02 | 7.93E-03 | -1.06 | down |
| GOLGA8A | 1.02E-01 | 4.01E-03 | -1.38 |  | 2.90E-02 | 1.70E-03 | -1.71 | down |
| PDE4D | 5.89E-02 | 3.90E-04 | -1.42 |  | 2.50E-02 | 9.94E-04 | -1.58 | down |
| NKTR | 5.46E-02 | 2.45E-04 | -1.42 |  | 9.09E-02 | 2.14E-02 | -1.05 | down |
| HSPA5 | 7.43E-02 | 1.21E-03 | -1.43 |  | 3.22E-02 | 2.30E-03 | -1.34 | down |
| HNRNPA2B1 | 5.01E-02 | 1.73E-04 | -1.43 |  | 6.57E-02 | 1.17E-02 | -1.29 | down |
| MGEA5 | 6.12E-02 | 4.23E-04 | -1.46 |  | 9.25E-02 | 2.20E-02 | -1.12 | down |
| SFPQ | 5.66E-02 | 3.10E-04 | -1.49 |  | 5.09E-02 | 7.12E-03 | -1.43 | down |
| ITPKB | 6.31E-02 | 5.49E-04 | -1.49 |  | 2.12E-02 | 5.52E-04 | -1.30 | down |
| N4BP2L2 | 6.14E-02 | 4.39E-04 | -1.49 |  | 3.57E-02 | 3.05E-03 | -1.20 | down |
| MPHOSPH6 | 7.82E-02 | 1.65E-03 | -1.53 |  | 6.04E-02 | 1.00E-02 | -1.29 | down |
| SRSF11 | 6.10E-02 | 4.20E-04 | -1.54 |  | 2.47E-02 | 9.66E-04 | -1.42 | down |
| HNRNPDL | 5.70E-02 | 3.19E-04 | -1.54 |  | 2.89E-02 | 1.65E-03 | -1.20 | down |
| CTSC | 1.59E-01 | 1.58E-02 | -1.65 |  | 8.02E-02 | 1.70E-02 | -1.49 | down |
| NTRK3 | 1.50E-01 | 1.33E-02 | -1.71 |  | 3.01E-02 | 1.91E-03 | -1.59 | down |
| IGHD | 6.76E-02 | 7.38E-04 | -3.60 |  | 3.19E-02 | 2.24E-03 | -2.61 | down |
